# Responses of Methanogenic and Methanotrophic Communities to Elevated Atmospheric CO_2_ and Temperature in a Paddy Field

**DOI:** 10.3389/fmicb.2016.01895

**Published:** 2016-11-24

**Authors:** Yuan Liu, Xiaoyu Liu, Kun Cheng, Lianqing Li, Xuhui Zhang, Jufeng Zheng, Jinwei Zheng, Genxing Pan

**Affiliations:** ^1^Department of Bioengineering, College of Life Science, Huaibei Normal UniversityHuaibei, China; ^2^Institute of Resources, Ecosystem and Environment of Agriculture, Nanjing Agricultural UniversityNanjing, China; ^3^Zhejiang Provincial Key Laboratory of Carbon Cycling in Forest Ecosystems and Carbon Sequestration, School of Environmental and Resource Sciences, Zhejiang A & F University, Lin’anHangzhou, China

**Keywords:** elevated CO_2_, warming, methanogen, methanotroph, paddy field

## Abstract

Although climate change is predicted to affect methane (CH_4_) emissions in paddy soil, the dynamics of methanogens and methanotrophs in paddy fields under climate change have not yet been fully investigated. To address this issue, a multifactor climate change experiment was conducted in a Chinese paddy field using the following experimental treatments: (1) enrichment of atmospheric CO_2_ concentrations (500 ppm, CE), (2) canopy air warming (2°C above the ambient, WA), (3) combined CO_2_ enrichment and warming (CW), and (4) ambient conditions (CK). We analyzed the abundance of methanogens and methanotrophs, community structures, CH_4_ production and oxidation potentials, *in situ* CH_4_ emissions using real-time PCR, T-RFLP, and clone library techniques, as well as biochemical assays. Compared to the control under CE and CW treatments, CH_4_ production potential, methanogenic gene abundance and soil microbial biomass carbon significantly increased; the methanogenic community, however, remained stable. The canopy air warming treatment only had an effect on CH_4_ oxidation potential at the ripening stage. Phylogenic analysis indicated that methanogens in the rhizosphere were dominated by *Methanosarcina, Methanocellales, Methanobacteriales*, and *Methanomicrobiales*, while methanotrophic sequences were classified as *Methylococcus, Methylocaldum, Methylomonas, Methylosarcina* (Type I) and *Methylocystis* (Type II). However, the relative abundance of *Methylococcus* (Type I) decreased under CE and CW treatments and the relative abundance of *Methylocystis* (Type II) increased. The *in situ* CH_4_ fluxes indicated similar seasonal patterns between treatments; both CE and CW increased CH_4_ emissions. In conclusion results suggest that methanogens and methanotrophs respond differently to elevated atmospheric CO_2_ concentrations and warming, thus adding insights into the effects of simulated global climate change on CH_4_ emissions in paddy fields.

## Introduction

Climate model projections suggest that atmospheric carbon dioxide (CO_2_) concentrations are likely to double by the end of the century, with mean global temperatures potentially increasing by a further 1.4–5.8°C (IPCC, 2007). Methane (CH_4_) is the second most abundant greenhouse gas (GHG) after CO_2_, accounting for about 20% of anthropogenic radiative forcing (Nisbet et al., 2014); atmospheric CH_4_ concentrations have increased from about 715 ppb before the industrial revolution to 1800 ppb in 2008 (Montzka et al., 2011). The global warming potential of CH_4_ is 25 times that of CO_2_, thus small changes in atmospheric CH_4_ concentrations will significantly contribute to future climate warming (Bridgham et al., 2013). Global climate change, such as elevated CO_2_ and warming, have been reported to dramatically alter the properties and functioning of terrestrial ecosystems (Rosenzweig et al., 2007; Austin et al., 2009; Singh et al., 2010).

One of the most important sources of atmospheric CH_4_ are rice paddies (Yan et al., 2009; Liu et al., 2012), accounting for 5–19% of global CH_4_ emissions (IPCC, 2007). An increase in CH_4_ emissions from these sources in response to elevated atmospheric CO_2_ and increased temperatures have already been identified, an occurrence which results in a positive feedback in the global warming process (Allen et al., 2003; Tokida et al., 2010; van Groenigen et al., 2011). Recent evidence has shown that elevated CO_2_ concentrations increased CH_4_ emissions from paddy soils by an average of 43% (van Groenigen et al., 2011); increased soil temperatures (2°C) resulted in a 42% increase in CH_4_ emissions (Tokida et al., 2010). It is generally assumed that elevated CO_2_ enhances photosynthesis, root biomass and exudates of rice (Pritchard, 2011; Okubo et al., 2014) which may provide more substrate for CH_4_ production (Inubushi et al., 2003). As methanogens and methanotrophs regulate CH_4_ emissions in rice soil (Conrad, 2007; Høj et al., 2008; Knoblauch et al., 2008), it is therefore important to understand how climate change factors affect microbial community structures and functions involved in the CH_4_ cycle.

CH_4_ production, the final microbial decomposition process of organic matter in paddy fields, is produced by methanogens (Bridgham et al., 2013; Breidenbach and Conrad, 2015), of which there are two main types of methanogenic pathways: acetate- and H_2_/CO_2_-dependent methanogenesis (Conrad and Klose, 2006; Breidenbach et al., 2015). Most biogenically produced methane is oxidized by methanotrophs at the soil surface (Conrad, 2007; Yun et al., 2013). CH_4_ oxidation can proceed both aerobically and anaerobically (Lüke et al., 2014; Knief, 2015). Aerobic methanotrophs are a subset of methylotrophs which can utilize CH_4_ as sole C and energy source (Chen et al., 2014). Aerobic methanotrophs in rice field consist mainly of proteobacterial lineages (Hu and Lu, 2015), while verrucomicrobial methanotrophs are restricted to extreme environments (Op den Camp et al., 2009). The proteobacterial methanotrophs can be separated into Type I and Type II groups belonging to *Gammaproteobacteria* and *Alphaproteobacteria*, respectively (Chen et al., 2014; Lee et al., 2014). Anaerobic methane oxidation can be coupled to sulfate reduction, metal reduction, nitrite dismutation, disulphide disproportionation (Ettwig et al., 2010; Joye, 2012). To study the diversity of methanogens and methanotrophs, we selected the genes coding for subunit A of the methyl coenzyme-M reductase enzyme (*mcrA*) and particulate methane monooxygenase enzyme (*pmoA*), respectively.

Methanogens have been identified to be sensitive to global climate change; atmospheric CO_2_ enrichment and warming alter the composition of methanogenic archaea and increase their abundance and activity in paddy soils (Peng et al., 2008; Liu et al., 2012). During a short-term incubation of paddy soil, due to reduced soil redox potential, increased available C and methanogens (Das and Adhya, 2012), elevated atmospheric CO_2_ and temperature interaction significantly increased CH_4_ production under flooded conditions. Elevated CO_2_ and increased carbon input from plants to soil may, have a positive effect on methanogenic archaea. However, Angel et al. (2012) identified that atmospheric CO_2_ enrichment had no significant impact on methanogenic community and CH_4_ production potential in a waterlogged grassland. On the other hand, CH_4_ oxidation decreased under elevated CO_2_ concentrations from different forest soils (McLain and Ahmann, 2008; Dubbs and Whalen, 2010), which may be due to increased soil moisture, the availability of carbon and reduced soil O_2_ concentrations under elevated CO_2_ conditions. Dijkstra et al. (2010) suggested that CH_4_ oxidation may be enhanced under drier soil conditions with increasing temperatures. However, studies investigating the responses of methanotrophic communities under paddy fields to elevated CO_2_ levels and atmospheric warming are limited.

While most experimental designs have studied the effects of a single climate factor (e.g., CO_2_ enrichment or increased temperatures) on soil CH_4_ cycling, the microbial responses in multi-factorial experiments have not been thoroughly investigated (Singh et al., 2010). In previous studies, elevated CO_2_ levels and atmospheric warming had either an additive or an antagonistic effect on soil microbial communities and functions in temperate agricultural soils (French et al., 2009; Pritchard, 2011). In order to assess how multiple climate change variables synergistically interact to affect soil methanogen and methanotroph microorganisms, we simultaneously artificially elevated atmospheric CO_2_ conditions (500 ppm, ambient) and air temperatures (+2°C, ambient) in a Chinese paddy field. The objective of this study was to address how CO_2_ enrichment, warming and their interaction affected the abundance and community composition of methanogens and methanotrophs in a rice paddy, and to determine if these changes could be linked to CH_4_ production and oxidization. The microbial abundance, community structure and composition were quantified and fingerprinted with real-time PCR (qPCR), terminal-restriction fragment length polymorphism (T-RFLP) and clone library techniques; CH_4_ production and oxidization potentials were assessed using biochemical assays.

## Materials and Methods

### Site Description and Experimental Setup

The field experiment simulating climate change was established in 2010 in Kangbo village (31°30′N, 120°33′E), Changshu Municipality, Jiangsu, China. The area experiences a subtropical monsoon climate with an annual mean temperature (2004–2013) of 16°C and mean precipitation of 1100–1200 mm. The soil is a Gleyic Stagnic Anthrosol formed on a clayey lacustrine deposit which has been cultivated with a rice-wheat rotation for hundreds of years. The basic properties of the topsoil prior to the experiment in 2010 were: soil pH of 7.0, organic carbon of 1.6%, total nitrogen of 1.9 g kg^-1^ and bulk density 12 g cm^-3^.

The experimental details with elevated CO_2_ and warming are presented in the study of Liu et al. (2014). In brief, with a block split-plot design, one field plot was artificially treated with a continuous atmospheric CO_2_ concentration enrichment up to 500 ppm (CE) using a liquid CO_2_ supply. The crop canopy air of another field plot was warmed by 2°C (WA) above the ambient temperature with infrared heaters. A further field plot was subjected to both CO_2_ enrichment and warming (CW), and a final field plot was maintained at an ambient condition as a control (CK). Treatment levels for our investigation were defined according to IPCC (2007): the A2 emission scenario predicts atmospheric CO_2_ concentrations to increase by 500 ppm and global mean air temperatures to increase by 2°C. For the elevated CO_2_ treatment, pure CO_2_ gas from a liquid tank was injected into the plots via perforated pipes surrounding the ring. Sixteen Li-820 CO_2_ sensors (Li-COR Inc., Lincoln, NE, USA) were installed and evenly distributed above the canopy to automatically control CO_2_ concentrations. CO_2_ concentration consistency over the ring was controlled by automatic adjustment to wind direction and velocity; when weed speed was more than 5 m/s, or if it was raining, CO_2_ spraying ceased. For the warming treatment, 12 infrared heaters (2000 W, 240 V, 1.65 m long × 0.14 m wide; HS-2420, Kalglo Electronics Co., Inc., Bethlehem, PA, USA) were situated on each ring. The heaters were adjusted every week to maintain a clearance height of 1.2 m above the top of the canopy during the growth period. The air temperature in the experimental plot was monitored by 6 infrared thermometers (Model SI- 121, Apogee instruments Inc., Logan, UT, USA) which were arranged in a hexagonal array (Liu et al., 2015; Cai et al., 2016; Wang et al., 2016). During the rice season, the average CO_2_ concentration under elevated CO_2_ plots was 515 ± 40 ppm and the increase of canopy air temperature under the warming plots was 1.98 ± 0.2°C. The control plots, surrounded by the same infrastructure, did not receive CO_2_ enrichment or any warming treatments. Each treatment was replicated in three rings with the same infrastructure, having 8-m-diameter and covering 50 m^2^ per ring. All the rings were buffered by an adjacent field to avoid treatment cross-over, and the distance between treatment plots was around 28 m.

In the experimental season, rice (*Oryza sativa* L. cv., Changyou No.5) was transplanted at a density of three seedlings per hill on 20th June, 2013. Plots were treated with local conventional practices, including a soil water regime of flooding during seedling to tillering stages, intermittent irrigation during heading, and drainage for ripening. Urea and ammonium bicarbonate were applied as basal fertilizers at a rate of 150 kg N ha^-2^ (120 kg-N ha^-2^ as urea and 30 kg-N ha^-2^ as ammonium bicarbonate) on 21th June, 2013. Chlorpyrifos was applied as a pesticide at a rate of 800–1000 g ha^-2^ at the heading stage. The management practices were consistent across all the treatments.

### Sample Collection

Rice rhizosphere soils were sampled at the tillering (19th July), heading (4th September) and ripening (24th October) stages in 2013. The rhizosphere of five individual rice plants were randomly collected at a depth of 0–15 cm from each plot, following the procedure described by Butler et al. (2003). The rhizosphere soil (being tightly adhered to the plant roots with about 1 cm thickness) was carefully removed and evenly mixed to form a composite sample. These soil samples were passed through a 2-mm sieve and immediately sealed in a plastic bag before being transferred to the laboratory (within 1 day after sampling). Fresh samples were stored at 4°C and analyzed for soil physico-chemical analyses within 1 week of sampling. A sub-sample of the soil was stored at –20°C prior to DNA extraction; this was undertaken within 1 week of sampling.

### Soil Property Analysis

Soil microbial biomass carbon (SMBC) was determined by a fumigation-extraction method following Wu et al. (1990). The samples were fumigated with ethanol free chloroform for 24 h at 25°C before being extracted with 0.5 mM K_2_SO_4_ for 30 min on a shaker; unfumigated samples were also processed using the same method. The extracts were analyzed for extractable C using an automated TOC Analyzer (TOC-500, Japan). A K_EC_ of 0.45 was used to convert the measured C to SMBC values. Inorganic N (NH_4_^+^ -N and NO_3_^-^ -N) was extracted by shaking with 0.5 mol L^-1^ K_2_SO_4_ (1: 5 (w/w) soil: K_2_SO_4_ solution) for 1 h and then filtering through a 0.45-um-pore-size polysulfone membrane, before colorimetric determination using an automated flow injection analyzer (Skalar Analytical B.V., The Netherlands).

### Measurement of Induced CH_4_ Production and Oxidation Potential

The CH_4_ production and oxidation potentials of soil were analyzed with a laboratory incubation method. CH_4_ production potentials in the soil samples were determined following the methods of Singh et al. (2012). In summary, 15 g of sample was transferred into a 120 ml glass jar, amended with 25 ml of sterile distilled deionized water and sealed with a butyl rubber stopper. The headspace in the jar was flushed with N_2_ for 10 min. Each soil sample was repeated in triplicate and incubated at 28°C in the dark. CH_4_ production was analyzed periodically by gas chromatography (Agilent 4890D, USA) equipped with a flame-ionization detector (FID). The CH_4_ concentration in the headspace was measured every 24 h for 1 week, and CH_4_ production potential was calculated using a linear regression of increased CH_4_ concentration with time.

CH_4_ oxidation potential was measured following the protocol of Vishwakarma et al. (2010). In summary, 15 g of sample was transferred into gas-tight 120 ml glass jars and incubated at 28°C for 7 days in the dark. All samples were analyzed in triplicate. The headspace in the jars contained 5% v/v methane in air. The CH_4_ concentration in the headspace was measured every 24 h for 1 week. CH_4_ oxidation potentials were calculated from the initial linear reduction of CH_4_ concentration with time and expressed as mg CH_4_-C per hour per gram dry weight.

### Monitoring CH_4_ Emissions

A static closed chamber-GS method was used to monitor CH_4_ flux (Zou et al., 2009). Gas samples were collected once a week during the rice growing season. Samples were collected between 08:00 and 10:00 on the collection day and 4 individual gas samples were collected with a syringe at 0, 10, 20, and 30 min after chamber closure. The concentration of CH_4_ in a sample was analyzed using a gas chromatograph (Agilent 7890A) equipped with a flame ionization detector (FID). The carrier gas was nitrogen and a flow rate of 40 ml/min was maintained. The oven and FID were operated at 50 and 300°C, respectively.

### DNA Extraction and Real-Time PCR Assay

Total DNA was extracted from 0.35 g of fresh soil with a PowerSoil^TM^ DNA isolation kit (MoBio, Carlsbad, CA, USA) following the manufacturer’s instructions. DNA quality was assessed on an agarose gel while DNA quantity was determined using a Nanodrop spectrophotometer (Thermo Scientific, DA, USA).

Real-time PCR was performed in a 7500 real-time PCR system (Applied Biosystems, Germany) via fluorometric monitoring with SYBR Green 1 dye. The primer pair mcrAF/mcrAR (Luton et al., 2002) and A189f/mb661r (Horz et al., 2001) were used to quantify methanogenic archaeal *mcrA* genes and methanotrophic bacterial *pmoA* genes of all samples, respectively. Each reaction was performed in a 25 μl volume containing 15 ng of DNA, 1 μl of 10 μM of each primer and 12.5 μl of SYBR premix EX Taq^TM^ (Takara Shuzo, Shinga, Japan). A melting curve analysis was conducted following each assay to confirm specific amplification was not from primer-dimers or other artifacts. A single clone containing the target region was grown in Luria-Bertani media and plasmid DNA was extracted using a plasmid-extraction kit (Takara, Japan). Standard curves were generated using a 10-fold dilution of plasmid DNA, from 10^3^ to 10^9^ copies of the template. PCR efficiencies were obtained between 98% and 106%, with *R*^2^ values >0.99. The final methanogenic *mcrA* gene and methanotrophic *pmoA* gene copy numbers were calibrated against total DNA amounts and soil water content.

### Terminal-Restriction Fragment Length Polymorphism Analysis of Soil Microbial Communities

Terminal-restriction fragment length polymorphism was used for analyzing the community structure of methanogens and methanotrophs. Briefly, the functional genes *mcrA* and *pmoA* were amplified by PCR using the primer pairs mcrAF/mcrAR and A189f/mb661r, as previously mentioned with the 5′ end of the mcrAF and A189f primers labeled with 6-carboxyfluorescein (6-FAM). All PCRs were performed in duplicate and pooled for subsequent restriction and T-RFLP analysis. PCR products were separated by 1.5% agarose gel, and purified using the PCR solution purification kit (Takara, Dalian, China). Purified PCR products were used in a restriction digest *Taq*I and *Msp*I (Takara, Dalian, China) for *mcrA* and *pmoA* genes as per the manufacturer’s instructions, respectively. Fragment analysis was achieved by capillary electrophoresis (ABI 3100 Genetic Analyzer; Applied Biosystems, Carlsbad, CA, USA) using a GeneScan ROX-labeled GS500 internal size standard. T-RFLP patterns were analyzed using GeneMapper software (Applied Biosystems) by peak height integration of different terminal restriction fragments (T-RFs). The fluorescence intensity (%) represented by a single T-RF was calculated relative to the total fluorescence intensity of all T-RFs. Peaks with heights that were less than 2% of the total peak height were excluded from further analysis to avoid potential noise before calculating relative T-RF abundance.

### Cloning, Sequencing, and Phylogenetic Analyses

Based on the obtained T-RFLP results, all soil samples at the ripening stage were chosen to establish clone libraries. Libraries for the functional genes *mcrA* and *pmoA* were created by ligating PCR products into pEASY-T3 vectors and being transformed into competent cells *Escherichia coli* JM109 (Takara, Japan) in accordance with the manufacturer’s instructions. Ninety four methanogenic clones and 102 methanotrophic clones were sequenced. All sequences were checked for chimera by using Bellerophon (Huber et al., 2004) before being grouped into operational taxonomic units (OTUs) using the furthest-neighbor clustering algorithm of the DOTUR software with a 96% threshold. *In silico* digests with *Taq*I and *Msp*I were undertaken on the sequences to allow the assignment of phylogenetic identity to individual peaks. The closest relatives of each sequence were checked using a BLAST search within GenBank. The representative sequences recovered in this study have been deposited in the GenBank database under accession numbers KU133526-KU133543 (methanogenic *mcrA* genes) and KU133544-KU133564 (methanotrophic *pmoA* genes).

### Statistical Analysis

Statistical analysis was performed using SPSS 20.0. One-way ANOVA with Tukey’s HSD test was used to test the difference among the treatments at each growth stage. Repeated measures ANOVA were used to determine the effect of climate change factors and plant growth stage on soil properties, CH_4_ production and oxidization rates, and microbial abundance (log_10_-transformed *mcrA* and *pmoA* gene abundances). Principal component analysis (PCA) of the T-RFLP profiles was performed using Minitab v.15 software based on relative fluorescence intensity of T-RFs. The probability level *p* < 0.05 was considered to be statistically significant.

## Results

### Soil Properties

Soil physico-chemical property data of the rice field soil under the simulated climate change conditions are shown in **Table 1**. Results showed that soil inorganic N (NH_4_^+^ and NO_3_^-^) did not significantly change under CE, CW, and WA treatments when compared with the control treatment. Soil NH_4_^+^ content generally declined with rice growth development across the treatments, ranging from 30.23 mg kg^-1^ (CW, tillering) to 7.88 mg kg^-1^ (CW, ripening). However, the content of NO_3_^-^ was stable without significant changes with the growth stages. Compared to CK, SMBC significantly increased under elevated CO_2_ levels (CE and CW) at all three growth stages, but the WA treatment significantly increased SMBC only at the heading stage. Repeated measures ANOVA showed that elevated CO_2_, warming and their combination significantly affected SMBC (*p* < 0.05), however, the interaction with the growth stage was not significant (**Table 2**).

**Table 1:** Variation in soil properties, CH_4_ production and oxidation potentials (mg kg^-1^ dw h^-1^) of the studied soils under climate change treatments.

Stage	Treatment	NH_4_^+^ (mg kg^-1^)	NO_3_^-^ (mg kg^-1^)	SMBC (mg kg^-1^)	CH_4_ production potential	CH_4_ oxidation potential
Tillering	CK	17.73 ± 5.46a	4.87 ± 0.45a	648.89 ± 38.74c	168.45 ± 17.22c	303.34 ± 27.79a
	CE	17.22 ± 4.36a	5.55 ± 0.61a	883.19 ± 96.67ab	214.22 ± 14.53ab	298.23 ± 21.12a
	CW	30.23 ± 7.13a	4.92 ± 0.15a	1031.90 ± 81.02a	225.00 ± 21.63a	325.78 ± 18.41a
	WA	17.90 ± 7.79a	4.89 ± 0.49a	762.81 ± 104.08bc	177.27 ± 27.07bc	324.13 ± 51.45a
Heading	CK	11.56 ± 1.05a	4.41 ± 0.87ab	518.87 ± 56.55c	205.43 ± 22.10b	191.62 ± 19.39a
	CE	14.15 ± 0.63a	5.40 ± 0.41a	823.37 ± 51.41ab	269.10 ± 25.62a	214.51 ± 19.70a
	CW	11.75 ± 0.29a	4.10 ± 0.28b	949.20 ± 33.49a	288.48 ± 24.21a	233.83 ± 26.11a
	WA	13.46 ± 2.32a	4.47 ± 0.39ab	727.47 ± 137.07b	197.42 ± 30.26b	247.08 ± 44.61a
Ripening	CK	11.12 ± 1.28a	3.90 ± 1.33a	608.77 ± 96.99b	141.14 ± 35.04b	328.44 ± 30.20b
	CE	8.53 ± 3.34a	4.80 ± 0.57a	960.04 ± 72.43a	187.55 ± 12.66a	359.42 ± 38.30b
	CW	7.88 ± 1.96a	4.78 ± 0.22a	930.04 ± 93.37a	171.10 ± 20.58ab	437.40 ± 20.71a
	WA	9.54 ± 1.45a	4.69 ± 0.21a	694.64 ± 91.14b	173.87 ± 17.03ab	473.77 ± 10.55a

*CK, ambient CO_*2*_ and ambient temperature; CE, atmosphere CO_*2*_ enrichment; CW, atmosphere CO_*2*_ enrichment and warming canopy air; WA, warming canopy air; SMBC: soil microbial biomass carbon. Data were presented as means of three replicates ± standard error; different letters within the same column indicate significant differences among treatments within a single growth stage (*p* < 0.05).*

**Table 2:** Repeated measures ANOVA for the effects of climate change, growth stage and their interaction on soil properties, CH_4_ production and oxidation potentials, abundances of *mcrA* and *pmoA* in the paddy soils under climate change treatments.

Stage	NH_4_^+^	NO_3_^-^	SMBC	CH_4_ production	CH_4_ oxidation	Methanogens abundance	Methanotrophs abundance
C	0.918	0.071	0.001	0.027	0.025	0.010	0.356
T	0.951	0.352	0.020	0.477	0.006	0.985	0.095
C × T	0.270	0.485	<0.001	0.016	0.001	0.010	0.133
S	0.008	0.320	0.072	<0.001	<0.001	<0.001	<0.001
C × S	0.407	0.938	0.385	0.540	0.973	0.048	0.642
T × S	0.674	0.642	0.562	0.353	0.004	0.837	0.063
C × T × S	0.023	0.403	0.426	0.141	0.065	0.129	0.080

*C: CO_*2*_ concentration; T: temperature; S: stage; SMBC: soil microbial biomass carbon.*

### CH_4_ Production and Oxidation Potentials, and CH_4_ Emissions

The CH_4_ production potentials ranged from 141.14 (CK, ripening) to 288.48 (CW, heading) mg CH_4_ kg^-1^ dw h^-1^ across the treatments and growth stages (**Supplementary Figure S2**). The highest values were recorded at the heading stage before a sharp decline at the ripening stage (**Table 1**). In contrary, CH_4_ oxidation potential declined at the heading stage before increasing to the highest values at the ripening stage (**Supplementary Figure S3**). Repeated measures ANOVA showed CO_2_ enrichment, warming and their interaction resulted in significant effects on CH_4_ oxidation potential, while elevated CO_2_ only affected CH_4_ production potential when crossed with warming (**Table 2**). Although potential CH_4_ production and oxidation potentials significantly differed among the growth stages, the effects of CO_2_ enrichment and warming did not depend on the growth stage. CH_4_ production potentials significantly increased under CO_2_ enrichment (CE and CW) treatments at all three growth stages, but no significant changes were observed under WA treatment or for the control. However, CH_4_ oxidation only increased at the ripening stage under warming treatments (33% for CW and 44% for WA).

The seasonal patterns of CH_4_ emission profiles were similar between the climate change treatments and the control (**Supplementary Figure S1**). As the paddy field was waterlogged, CH_4_ concentration peaks occurred 20–35 days after transplanting. After 35 days, CH_4_ emissions sharply declined and remained at a low rate until harvest. Elevated CO_2_ significantly increased CH_4_ emissions in this paddy field. Compared to the CK treatment, mean CH_4_ emissions increased by 37% and 25% under CE and CW treatments, respectively.

### Abundance of Methanogens and Methanotrophs

**FIGURE 1:**
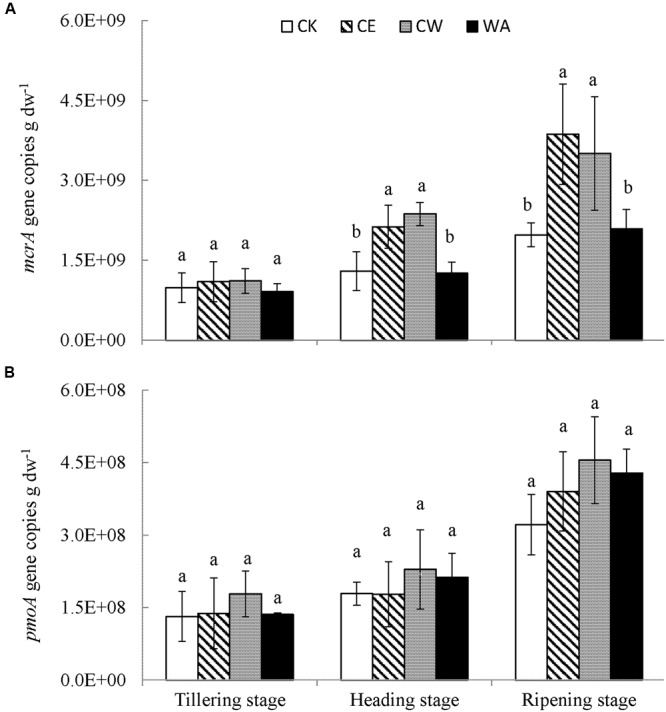
**Changes in the *mcrA* (A)** and *pmoA* **(B)** gene copy numbers for methanogens and methanotrophs in the studied soils under simulated climate change treatments. Different letters above the columns indicate significant differences among treatments within a single growth stage (*p* < 0.05).

Gene abundance data showed that the abundances of *mcrA* and *pmoA* genes generally increased with rice growth development, the highest values being attained at the ripening stage (**Figure 1**). The abundance of *mcrA* genes under all treatments ranged from 9.13 × 10^8^ (WA, tillering) to 7.73 × 10^9^ (CE, ripening) copies g^-1^ dw. These results were higher than the abundance of *pmoA* genes, ranging from 1.31 × 10^8^ (CK, tillering) to 4.55 × 10^8^ (CW, ripening) copies g^-1^ dw. Repeated measures ANOVA showed that the effects of elevated CO_2_ and elevated CO_2_ combined with warming were significant (*p* < 0.05) on the abundance of *mcrA* genes, but not on the abundance of *pmoA* genes (**Table 2**). In this study, no significant changes in the abundance of *mcrA* and *pmoA* genes associated with climate change treatments were observed at the tillering stage. However, the abundance of *mcrA* genes significantly increased under CE and CW treatments at the heading and ripening stages. Compared to CK, the mean abundance of *mcrA* genes increased by 63% (CE) and 82% (CW) at the heading stage and 98% (CE) and 78% (CW) at the ripening stage, respectively. In contrast, the *pmoA* gene copy numbers were more stable without significant changes among the climate change treatments at all three growth stages.

### Structure Composition of Methanogens and Methanotrophs

**FIGURE 2:**
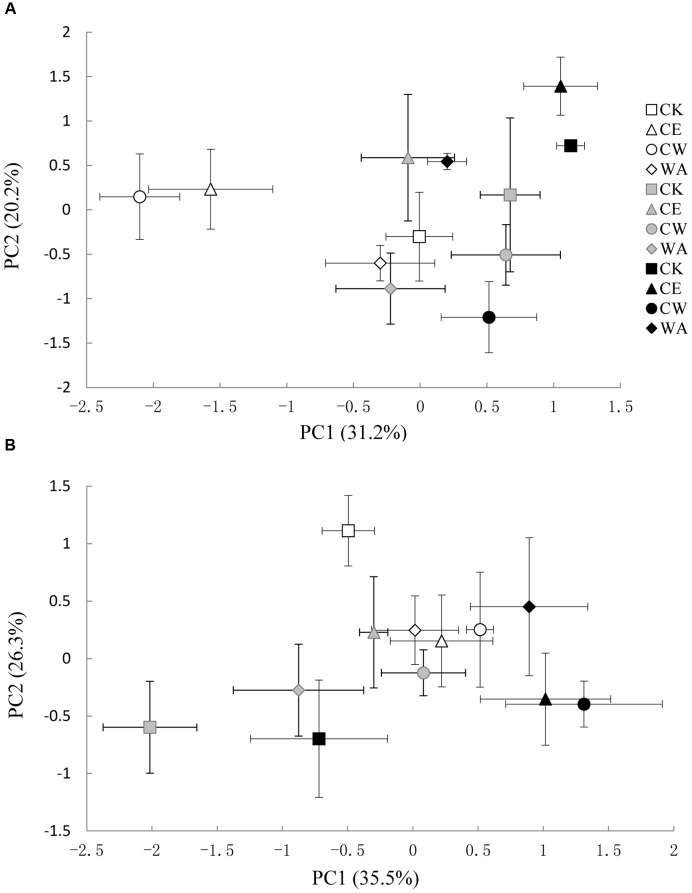
**Principal component analysis (PCA) of T-RFLP patterns of methanogens (A)** and methanotrophs **(B)** from the studied soils. Tillering stage (white); Heading stage (light gray); Ripening stage (black). The symbols are as follows: ambient CO_2_ and ambient temperature (CK), squares; atmosphere CO_2_ enrichment (CE), triangles; atmosphere CO_2_ enrichment and warming canopy air (CW), circles; warming canopy air (WA), diamonds. The error bars indicate the standard error of the means.

The methanogenic and methanotrophic community structures were analyzed using T-RFLP fingerprints. PCA of the T-RFLP profiles at the three growth stages yielded summaries of data, as 51.4% for *mcrA* genes and 61.8% for *pmoA* genes of the total variability was explained by PC1 and PC2 (**Figure 2**). No clear differences in the methanogenic community structure between the treatments and across the growth stages were highlighted by PCA analysis. **Figure 2B** shows that the methanotrophic community structure under CE, CW, and WA was distinctively separated from the control at each stage.

**FIGURE 3:**
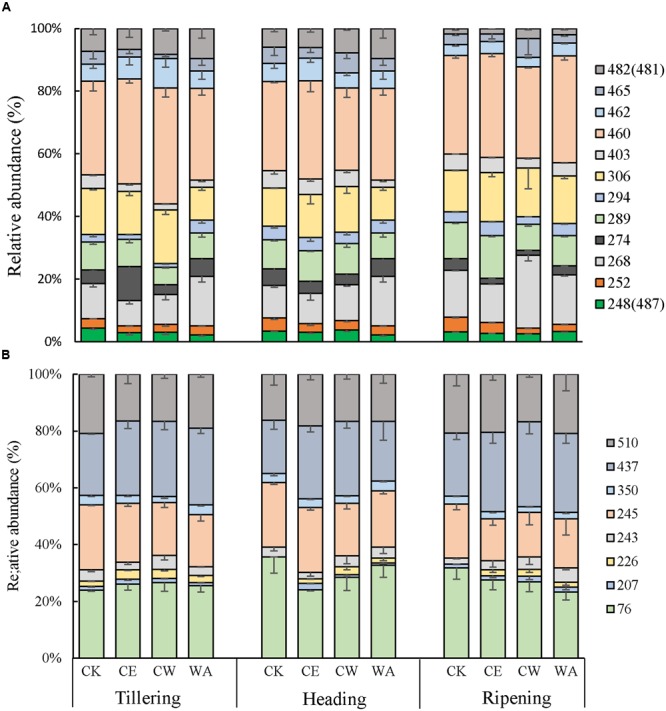
**Relative abundance of T-RFs for *mcrA* (A)** and *pmoA* **(B)** genes as determined by T-RFLP analysis in the studied soils. CK, ambient CO_2_ and ambient temperature; CE, atmosphere CO_2_ enrichment; CW, atmosphere CO_2_ enrichment and warming canopy air; WA, warming canopy air. Only major T-RFs (reductive abundance >1%) are shown. The error bars indicate the standard error of the means (*n* = 3).

A total of 12 and 8 T-RFs were obtained from the overall samples for *mcrA* and *pmoA* genes, respectively. T-RFLP fingerprinting of *mcrA* genes revealed that the 268bp, 289bp, 306bp, and 460bp TRFs were dominant in all treatments, while T-RFs of 76bp, 245bp, 437bp, and 510bp were dominant in the methanotrophic *pmoA* T-RFLP profiles (**Figure 3**). The main T-RFs in the T-RFLP profiles were identified with *in silico* restriction of clone sequences. T-RFs related to *Methanosarcina* were the most predominant (35–45%) across all treatments while T-RFs related to *Methanocellales* and *Methanobacteriales* ranged from 19–29% and 13–28%, respectively. The effect of simulated climate change scenarios on the relative abundance of *mcrA* TRFs was insignificant. However, the relative abundance of the 76bp TRF, related to *Methylococcus*, decreased under CE and CW treatments at the later growth stages, while that of the 437bp TRF related to *Methylocystis* increased.

**FIGURE 4:**
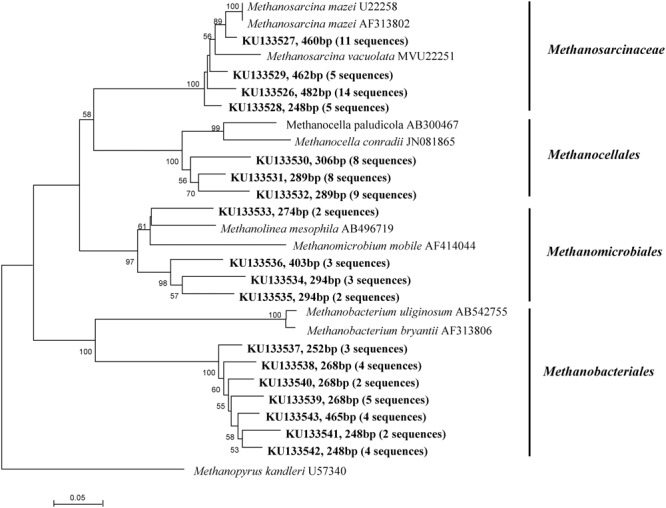
**Phylogenetic tree of representative methanogenic sequences retrieved from the rhizosphere samples of paddy soil and reference sequences from GenBank.** Bootstrap values of >50% are indicated at branch points. The accession number and terminal restriction fragment (T-RF) sizes digested *in silico* are shown in bold.

**FIGURE 5:**
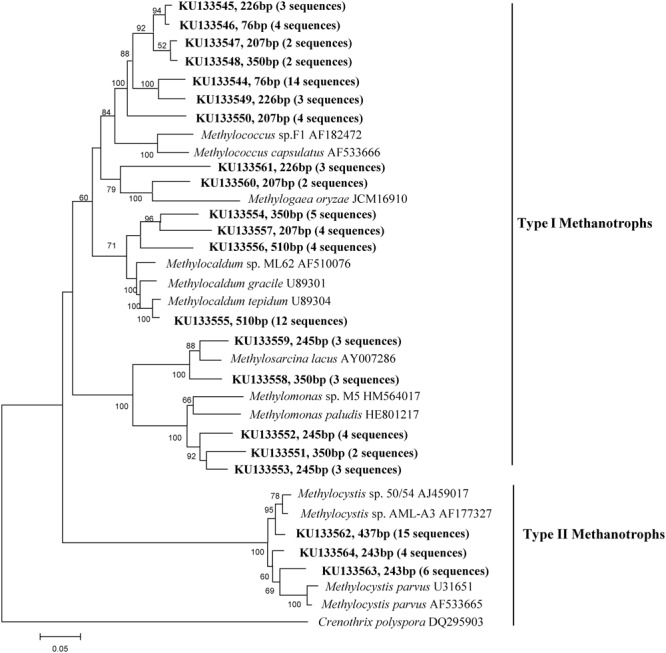
**Phylogenetic tree of representative methanotrophic sequences retrieved from the rhizosphere samples of paddy soil and reference sequences from GenBank.** Bootstrap values of >50% are indicated at branch points. The accession number and terminal restriction fragment (T-RF) sizes digested *in silico* are shown in bold.

The clone library analysis of *mcrA* and *pmoA* yielded a total of 94 and 102 cloned *mcrA* and *pmoA* sequences, respectively. Phylogenetic analyses of the *mcrA* sequences revealed that the methanogenic community in this paddy soil was dominated by members of *Methanosarcina, Methanocellales, Methanobacteriales*, and *Methanomicrobiales* (**Figure 4**). Twenty-one different OTUs of *pmoA* sequences were confirmed (**Figure 5**). The phylogenetic pattern of methanotrophic clones indicated that *Methylococcus, Methylocaldum, Methylomonas, Methylosarcina, Methylogaea* (Type I), and *Methylocystis* (Type II) were generally dominant during the rice growth period. As shown in **Figure 5**, *Methylococcus* (7 OTUs) and *Methylocaldum* (4 OTUs) sequences were the most dominant in Type I, representing about 31 and 25% of total clones in the *pmoA* clone library, respectively. To a smaller extent, Type I sequences were affiliated with *Methylomonas* (9 sequences), *Methylosarcina* (6 sequences) and *Methylogaea* (5 sequences). *Methylocystis* (3 OTUs, 25 sequences), the only groups of Type II methanotrophs, were identified in the rhizosphere.

## Discussion

### Methane Production Potential and Methanogen Community

Methanogenesis favors anoxic conditions with a low redox potential and a neutral pH in submerged soil (Masscheleyn et al., 1993; Orphan et al., 2001). In a laboratory incubation study, Das and Adhya (2012) found that elevated CO_2_ significantly increased CH_4_ production which was highly associated with decreased soil redox potential and pH in paddy soils. In this study, it was observed that CO_2_ enrichment significantly increased CH_4_ production potential during the rice growth period, whereas much higher SMBC was found (**Table 1**). This finding was consistent with results from Das et al. (2011) who observed an increase in SMBC with elevated CO_2_ concentrations and temperatures in a rice soil incubation experiment. Das and Adhya (2012) noted that an increase of methanogenic population with elevated CO_2_ levels was the most important reason for enhanced CH_4_ production under CO_2_ enrichment. In a free air CO_2_ enrichment (FACE) experiment, Okubo et al. (2015) also found that the number of copies of the *mcrA* gene significantly increased when CO_2_ concentrations were elevated in rice paddy fields. Similarly, our results showed that elevated CO_2_ concentrations significantly increased the abundance of methanogens in the rice rhizosphere soil (**Figure 1**). In this experiment, photosynthesis and total biomass of rice increased with elevated CO_2_ (Cai et al., 2016; Wang et al., 2016), resulting in an increase in root exudates and rhizodeposition in the rhizosphere (Bhattacharyya et al., 2013; Okubo et al., 2014). Furthermore, rhizodeposition is regarded as the primary source of CH_4_ produced in rice fields (Conrad, 2007). Increased carbon inputs into the rhizosphere soil could also increase microbial biomass and activity (Drissner et al., 2007; French et al., 2009).

Based on T-RFLP and clone sequence analyses, we found that methanogens belonging to *Methanosarcina, Methanobacteriales*, and *Methanocellales* (Rice Cluster I) were dominant in this paddy soil. A similar result was found in other studies focusing on methanogenic diversity of paddy soils (Liu et al., 2012; Breidenbach et al., 2015). *Methanosarcina*, the only species of acetoclastic groups, were present during the rice growth period, suggesting that acetate may be a major substrate for CH_4_ production in paddy fields. This observation is consistent with previous investigations showing *Methanosarcina* as a major species in rice fields under both flooded and drained conditions (Watanabe et al., 2009; Itoh et al., 2013). Based on RNA stable isotope probing analysis, Lu and Conrad (2005) indicated that individuals of the species *Methanocellales* were the most active for metabolizing rice root exudates, and that they play an important role in CH_4_ production in paddy fields. In this study, elevated CO_2_ and warming did not significantly alter the relative abundance of methanogenic T-RFs, resulting in minor changes in the composition of the methanogenic community. Thus, it is possible that the increase in CH_4_ production with elevated atmospheric CO_2_ is driven by the accumulation of substrate input and an increase in the methanogenic population or cell-specific activity without shifting methanogenic community composition.

In our study, a 2°C increase in air temperature did not change the activity, abundance or community composition of methanogens compared with elevated CO_2_ treatments. This finding differs from the findings of Das and Adhya (2012); they identified an increase in CH_4_ production and methanogenic population in trophic paddy soils associated with a temperature increase from 25–45°C, at intervals of ten degrees. Generally, an increase in temperature stimulates the decomposition of organic matter in submerged soils (von Lützow and Kögel-Knabner, 2009; Karhu et al., 2010) which may lead to higher rates of CH_4_ production under anaerobic conditions (Fey and Conrad, 2000). In this study, the 2°C increase in canopy air temperature resulted in a small increase in soil temperature (<1°C, unpublished data), an increase which is in the range of daily/seasonal fluctuations and heterotrophic microbial communities are insensitive to such temperature increases (Malchair et al., 2010). Moreover, the direct effects of warming on soil microbial communities could be confounded by soil moisture content, substrate availability and plant conditions (Allison and Treseder, 2008; Das and Adhya, 2012).

### Methane Oxidation Potential and Methanotroph Community

Previous studies indicated that CH_4_ oxidation potential increased as soil temperatures increased (Van den Pol-van Dasselaar et al., 1998; Dijkstra et al., 2010). Here, we showed that warming treatments (CW and WA) resulted in significant increases in CH_4_ oxidation potential at the ripening stage when the paddy field was drained, whereas no change was observed under CE during the rice growth period. In a ^13^C-labeling study, Kalyuzhanaya et al. (2013) observed that a significantly larger portion of the assimilated carbon (over 62%) was derived from CO_2_ in the alphaproteobacterial methanotrophs. Autotrophic methanotrophs, potentially utilizing C-1 compounds for their metabolic activity, could be greatly affected by changing environmental conditions. For example, if an increase of temperature results in the soil becoming drier, CH_4_ oxidation may be enhanced (Dijkstra et al., 2010). Furthermore, as well as temperature, the water-logging regime is the other most important factor for CH_4_ oxidation in rice fields (Das and Adhya, 2012). In the present study, such an increase of CH_4_ oxidation potential under warming treatments was greater at the ripening stage (by 33–45%) when the rice fields were drained than at the tillering and heading stages (by 7–28%) when they were flooded. The growth stage of rice could therefore play an important role in the abundance and activity of methanotrophs during the rice growth period (Eller and Frenzel, 2001; Lüke et al., 2010; Lee et al., 2014). In a study of rice paddy soil, Ho et al. (2011) found that the higher methane oxidation potential related well to the cell-specific activity and population of methanotrophs. Though a nitrite-driven anaerobic CH_4_ oxidation was discovered in wetlands (Hu et al., 2014), aerobic methanotrophs in rice field were predominant (Hu and Lu, 2015).

The composition and distribution of methanotrophs were relatively stable across the simulated climate change treatments. In this study, *Methylococcus* and *Methylocaldum* (both Type I), and *Methylocystis* (Type II), were dominant in the rice rhizosphere (**Figures 4** and **5**). Similar data have been found in rice field soils from Aichi-ken Anjo Research and Extension Center, central Japan (Jia et al., 2007), Gangetic plain of India (Vishwakarma et al., 2010) and National Rice Research Institute of China (Ma et al., 2010). It is generally assumed that Type I methanotrophs to be highly responsive to high substrate resources (Ho et al., 2013; Chen et al., 2014) while Type II methanotrophs are relatively stable (Eller et al., 2005; Krause et al., 2012). As an indication with a fast growth rate, Type I methanotrophs were predominant in occupying niches with abundant resources (Ho et al., 2013), such as in the rhizosphere and on the roots of rice plants (Wu et al., 2009; Chen et al., 2014). In this study, it is possible that increased labile organic compounds under warming are not large enough to affect the population or composition of the Type I methanotrophs.

## Conclusion

In this study, we observed increased CH_4_ production potential at the three rice stages in response to atmospheric CO_2_ enrichment. An increase in the methanogenic population is the most likely cause of increased CH_4_ production under elevated CO_2_ conditions. Warming treatments resulted in a significant increase in CH_4_ oxidation potential at the ripening stage, without any change in the abundance and community composition of methanotrophs during the growth period. Our data demonstrate that methanogens and methanotrophs differentially responded to elevated atmospheric CO_2_ and warming. Results from our investigation will enable a more comprehensive understanding of the future role of microbial processes and related microorganisms in methane emissions. However, future research should also investigate the long-term interactive effects of elevated atmospheric CO_2_ and warming on the carbon cycle under field conditions.

## Author Contributions

GP and LL designed research; YL and XL performed the data analysis. YL wrote the paper. KC, XZ, and JFZ revised and commented on the draft. All authors read and approved the final manuscript.

## Conflict of Interest Statement

The authors declare that the research was conducted in the absence of any commercial or financial relationships that could be construed as a potential conflict of interest.

## Supplementary Material

FIGURE S1 **CH_4_ flux dynamics during the rice growing season.** The symbols are as follows: ambient CO_2_ and ambient temperature (CK), squares; atmosphere CO_2_ enrichment (CE), triangles; atmosphere CO_2_ enrichment and warming canopy air (CW), circles; warming canopy air (WA), diamonds. The error bars indicate the standard error of the mean (*n* = 3).

FIGURE S2 **CH_4_ production potentials (mg g^-1^ dw h^-1^) during the incubation.** Tillering stage **(A)**; Heading stage **(B)**; Ripening stage **(C)**. The symbols are as follows: ambient CO_2_ and ambient temperature (CK), squares; atmosphere CO_2_ enrichment (CE), triangles; atmosphere CO_2_ enrichment and warming canopy air (CW), circles; warming canopy air (WA), diamonds. The error bars indicate the standard error of the mean (*n* = 3).

FIGURE S3 **CH_4_ oxidation potentials (mg g^-1^ dw h^-1^) during the incubation.** Tillering stage **(A)**; Heading stage **(B)**; Ripening stage **(C)**. The symbols are as follows: ambient CO_2_ and ambient temperature (CK), squares; atmosphere CO_2_ enrichment (CE), triangles; atmosphere CO_2_ enrichment and warming canopy air (CW), circles; warming canopy air (WA), diamonds. The error bars indicate the standard error of the mean (*n* = 3).
