# Supplementary figures and images for: Responses of Methanogenic and Methanotrophic Communities to Elevated Atmospheric CO2 and Temperature in a Paddy Field
Source: Front Microbiol. 2016 Nov 24;7:1895. doi: 10.3389/fmicb.2016.01895 (PMC5121223; doi:10.3389/fmicb.2016.01895)

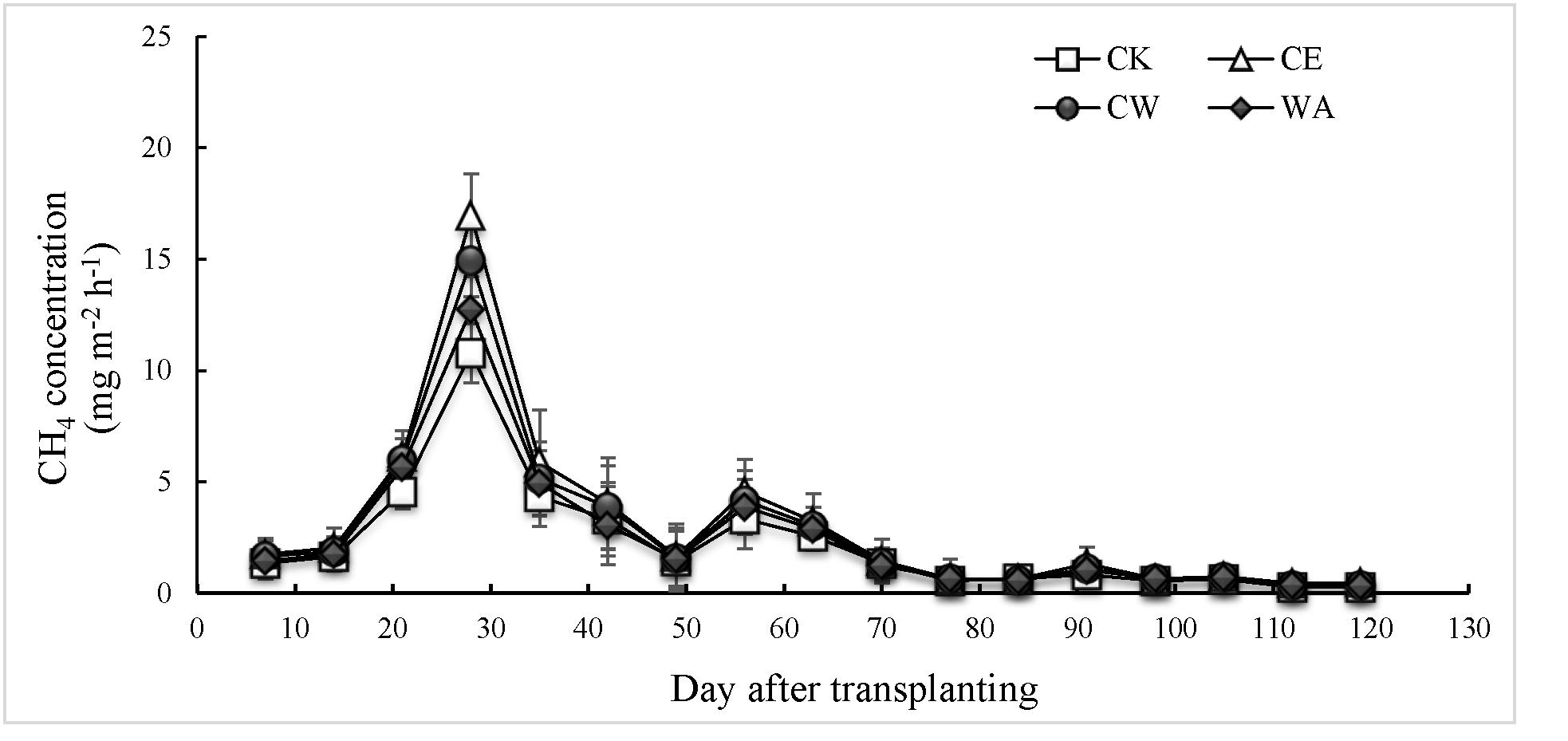

Supplement: FIGURE S1 — CH4 flux dynamics during the rice growing season. The symbols are as follows: ambient CO2 and ambient temperature (CK), squares; atmosphere CO2 enrichment (CE), triangles; atmosphere CO2 enrichment and warming canopy air (CW), circles; warming canopy air (WA), diamonds. The error bars indicate the standard error of the mean (n = 3). [file Image_1.TIFF]

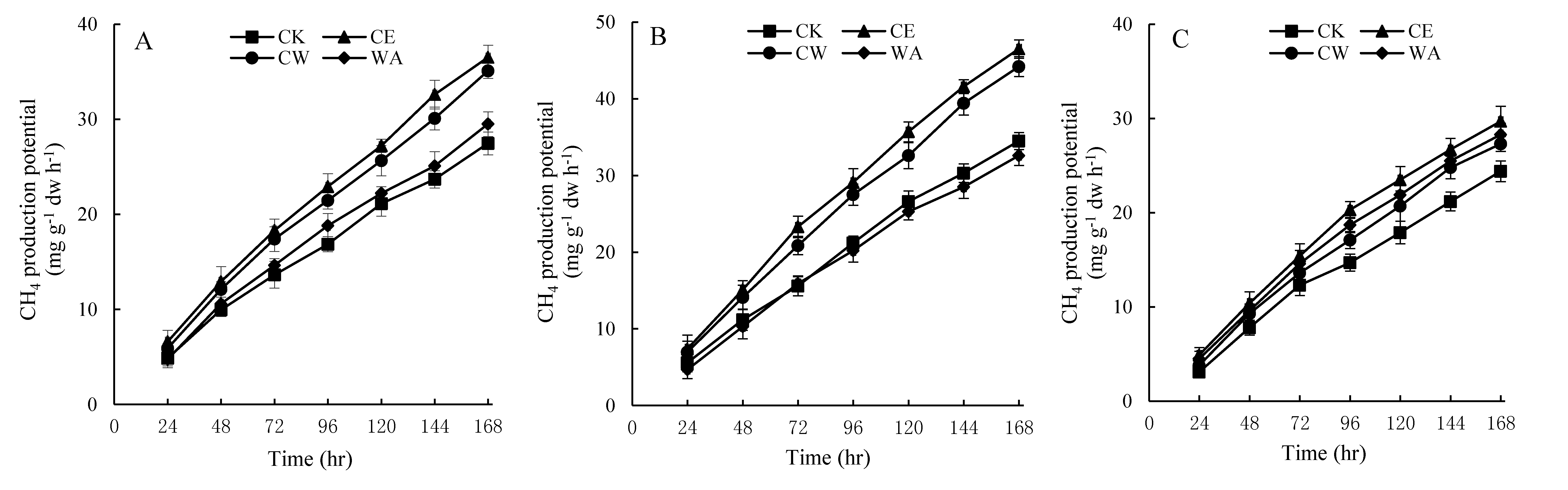

Supplement: FIGURE S2 — CH4 production potentials (mg g-1 dw h-1) during the incubation. Tillering stage (A); Heading stage (B); Ripening stage (C). The symbols are as follows: ambient CO2 and ambient temperature (CK), squares; atmosphere CO2 enrichment (CE), triangles; atmosphere CO2 enrichment and warming canopy air (CW), circles; warming canopy air (WA), diamonds. The error bars indicate the standard error of the mean (n = 3). [file Image_2.TIFF]

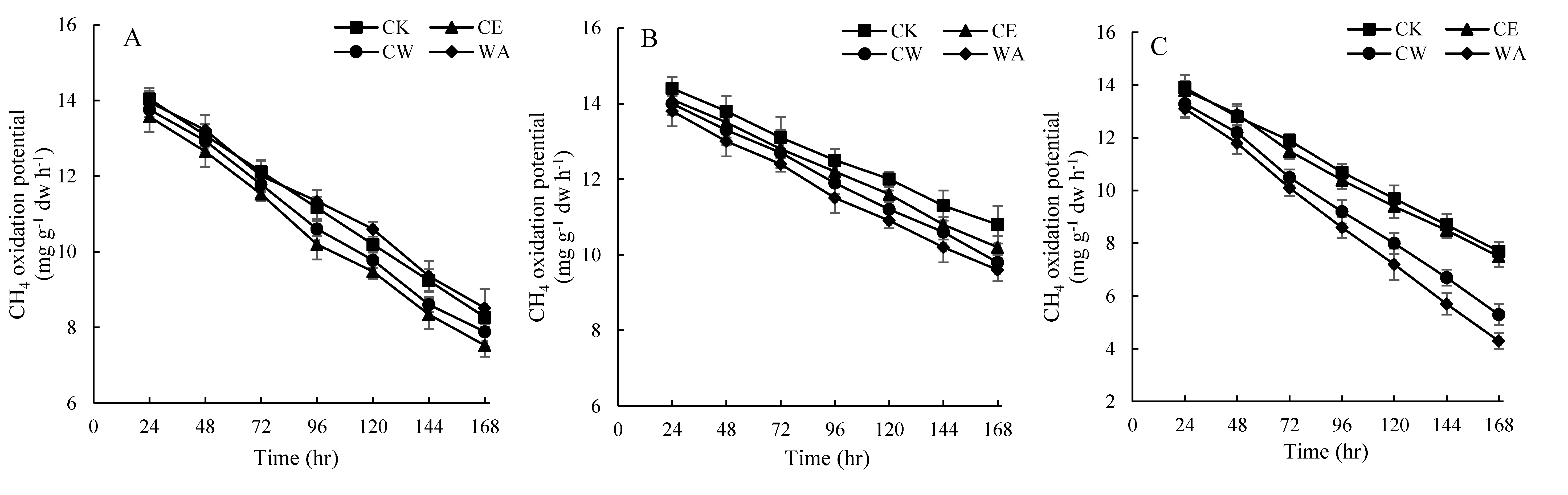

Supplement: FIGURE S3 — CH4 oxidation potentials (mg g-1 dw h-1) during the incubation. Tillering stage (A); Heading stage (B); Ripening stage (C). The symbols are as follows: ambient CO2 and ambient temperature (CK), squares; atmosphere CO2 enrichment (CE), triangles; atmosphere CO2 enrichment and warming canopy air (CW), circles; warming canopy air (WA), diamonds. The error bars indicate the standard error of the mean (n = 3). [file Image_3.TIFF]
